# A serine–arginine-rich (SR) splicing factor modulates alternative splicing of over a thousand genes in *Toxoplasma gondii*

**DOI:** 10.1093/nar/gkv311

**Published:** 2015-04-13

**Authors:** Lee M. Yeoh, Christopher D. Goodman, Nathan E. Hall, Giel G. van Dooren, Geoffrey I. McFadden, Stuart A. Ralph

**Affiliations:** 1 Department of Biochemistry and Molecular Biology, Bio21 Molecular Science and Biotechnology Institute, The University of Melbourne, Parkville, Victoria 3010, Australia; 2 School of BioSciences, The University of Melbourne, Parkville, Victoria 3010, Australia; 3 Department of Genetics, La Trobe Institute for Molecular Science, La Trobe University, Bundoora, Victoria 3086, Australia; 4 Life Sciences Computation Centre, Victorian Life Sciences Computation Initiative, Carlton, Victoria 3010, Australia; 5 Research School of Biology, The Australian National University, Acton, ACT 2601, Australia

## Abstract

Single genes are often subject to alternative splicing, which generates alternative mature mRNAs. This phenomenon is widespread in animals, and observed in over 90% of human genes. Recent data suggest it may also be common in Apicomplexa. These parasites have small genomes, and economy of DNA is evolutionarily favoured in this phylum. We investigated the mechanism of alternative splicing in *Toxoplasma gondii*, and have identified and localized TgSR3, a homologue of ASF/SF2 (alternative-splicing factor/splicing factor 2, a serine-arginine–rich, or SR protein) to a subnuclear compartment. In addition, we conditionally overexpressed this protein, which was deleterious to growth. qRT-PCR was used to confirm perturbation of splicing in a known alternatively-spliced gene. We performed high-throughput RNA-seq to determine the extent of splicing modulated by this protein. Current RNA-seq algorithms are poorly suited to compact parasite genomes, and hence we complemented existing tools by writing a new program, GeneGuillotine, that addresses this deficiency by segregating overlapping reads into distinct genes. In order to identify the extent of alternative splicing, we released another program, JunctionJuror, that detects changes in intron junctions. Using this program, we identified about 2000 genes that were constitutively alternatively spliced in *T. gondii*. Overexpressing the splice regulator TgSR3 perturbed alternative splicing in over 1000 genes.

## INTRODUCTION

The phylum Apicomplexa includes the most important eukaryotic pathogens of humans, including *Plasmodium falciparum*, causative agent of severe human malaria, and *Toxoplasma gondii*, causative agent of toxoplasmosis. Apicomplexan parasites cause millions of infections and diverse human and animal diseases. Malaria is predominantly a disease of poverty, with almost half of the world's population at risk, resulting in over half a million deaths every year (World Health Organization, http://www.who.int/features/factfiles/malaria). Toxoplasmosis is primarily of concern to immunocompromized individuals and pregnant women.

The success of eukaryotic pathogens can be partially attributed to their sophisticated gene regulation. In particular, the varied life stages of apicomplexan parasites demand tight regulation of transcription and translation. Abundant experimental data demonstrate that transcription of most genes is exquisitely tuned to parasite development (1). This includes temporal regulation within discrete life-cycle stages, such as the ring to trophozoite forms within the *Plasmodium* erythrocytic proliferative stage, and between the major life stages, such as the *Plasmodium* liver to erythrocytic stages, or the *T. gondii* acute-disease causing tachyzoites to cyst-forming bradyzoites. Compounds that interfere with transcriptional regulation lead to death or growth arrest, or may interfere with stage progression (2), and may have potential as anti-malaria drugs (3,4).

Alternative splicing is the production of more than one splice variant from a single gene. This phenomenon can result in exon skipping, intron retention, mutually-exclusive exons or changes to a single intronic splice site. Alternative splice forms may generate proteins with different molecular functions, different structures and different sub-cellular localizations, thus impacting cellular function (5). Apicomplexans have small genomes, and economy of DNA appears to be evolutionarily favoured for this phylum (6). Thus, alternative splicing may be an important means by which to maintain transcriptional complexity within the limits of their compact genomes.

Understanding alternative splicing is important for two reasons. First, we need to appreciate the richness of transcript complexity in order to have a thorough idea of what gene products actually exist in cells. Only a very small number of alternatively spliced genes have been investigated in *Plasmodium* and *Toxoplasma*, but these already amply demonstrate the limitations of understanding any protein's function without full information about splice forms. These genes include the cysteinyl tRNA synthetase (7,8), ALAD/SPP (9), myosin B/C (10), HXGPRT (11) and some surface protein such as MAEBL, stevor and yir (12–14). Many of these alternatively-spliced products are putative targets for vaccines or drugs, so understanding the diversity of these protein products is central to combating parasitic diseases.

A second compelling reason to study alternative splicing is that this process presents an attractive drug target in its own right. Several compounds that modulate alternative splicing are already clinically-used drugs, and have been proposed as treatments for fungal infections, cancers and viral infections (15–18). Molecular targets in alternative splicing include the spliceosome itself, effectors of alternative splicing, and protein kinases that phosphorylate these effectors (16). Several existing inhibitors of kinases acting on alternative-splicing factors are selectively toxic to tumour cells (15,19), validating the use of such compounds in humans.

In metazoans, high-throughput sequencing reveals an abundance of alternative splicing. For example, RNA-seq analysis of ten different tissues and five mammary cancer cell lines showed that 90% of human genes were alternatively spliced (20). Most alternative splice forms show tissue specificity, indicating that diversity in splice forms contributes to the differences between cell types (20,21). Despite the importance of this process, we have little understanding of the role of alternative splicing in any pathogen, including the important disease-causing apicomplexans. Differences in transcript abundance between parasite life stages are comparable to changes seen between different cell types of metazoans (22) so it is also reasonable to infer that differences in splice forms in apicomplexans will be regulated between life stages. We hypothesize that the diversity of alternative splicing that occurs between cell types in multicellular organisms will be reflected in diversity of splicing between life stage cell types of the unicellular Apicomplexa and will be crucial to the biology of infection.

Eukaryotic canonical splicing of protein-coding genes is carried out by the spliceosome machinery, an assembly of proteins and snRNAs that bind to and remove intronic sequences in mRNA (23). The recognition of introns by the spliceosome is regulated by a large number of protein factors. One important mediator of alternative splicing is the alternative-splicing factor/splicing factor 2 (ASF/SF2 or SRSF1) from the serine/arginine-rich (SR) family.

The precise mechanism of SR proteins in alternative splicing is poorly understood, but it is thought that these proteins enhance or repress the association of spliceosomal components with splice sites, depending on the SR protein's binding position with RNA (24). SR proteins can thereby enhance or inhibit alternative splicing of specific mRNA species. SR proteins localize to subnuclear compartments known as speckles; these consist of interchromatin granule clusters, where splicing factors are assembled and/or stored and perichromatin fibrils, where RNA is transcribed and concurrent splicing of pre-RNA probably occurs (25). These speckles are distinct from the nucleolus and condensed chromatin (26).

SR proteins have roles in constitutive splicing, mRNA export, conferring mRNA stability and translation regulation (27). However, knockdown or overexpression of SF2/ASF has been used to perturb alternative splicing specifically, resulting in a concomitant change in the proportion of selected mRNA isoforms as detected by qRT-PCR (28). The role of the other SR proteins in alternative splicing has not been experimentally validated, although upregulation of some of these proteins correlates with cancer in metazoans, which is often associated with incongruent alternative splicing (28).

There has been a paucity of research into apicomplexan SR proteins and their downstream targets. A recent study identified an SR-related protein in *P. falciparum* that exhibited alternative-splicing activity in an *in-vitro* assay, and of exogenous reporter genes *in vivo* (29). Overexpression of this protein resulted in a reduction in parasite proliferation and a small-scale qRT-PCR–based screen revealed changes in alternative splicing of three genes (29).

In this study, we identify four SR proteins in *T. gondii*, and overexpress one of these to determine the transcripts perturbed by this alternative-splicing factor. Previously, the number of genes alternatively spliced in apicomplexans has been severely under-reported (30–33), and we partially address this by identifying alternatively-spliced genes modulated by this SR protein. This type of survey has only recently become technologically feasible, with previous transcriptomic analyses relying on microarrays, which suffer from technical limitations that prevent reliable identification of alternative splicing.

## MATERIALS AND METHODS

### Phylogenetic analyses

Sequences for the twelve known human SR proteins (34,35) were obtained from Genbank (36) and used as bait to identify homologues in *P. falciparum*, *T. gondii* and *Arabidopsis thaliana*, using a combination of OrthoMCL mining (37) (for the apicomplexans and *A. thaliana*), and BLAST homology searches in PlasmoDB, ToxoDB and the Genbank nr database (for the apicomplexans) or the Genbank refseq_protein database (for *A. thaliana*) (36,38,39). Results from BLAST homology searches were only included in downstream analyses if the reciprocal best hit against the Genbank human nr database matched with an SR protein. Sequences were then aligned using Clustal Omega with default options (40), and manually aligned and trimmed with Geneious Pro (41). Well-aligning regions were used for phylogenetic tree construction, which correlated with the N-terminal RNA-recognition motif. Alternatively-spliced homologues were initially included; however, isoforms with redundancy within the inclusion set were removed, selecting the best-aligning protein when relevant, and the remaining proteins realigned and trimmed. A maximum-likelihood tree was constructed using PhyML with 100 bootstrap replicates, using the JTT substitution model, an estimated proportion of invariable sites, and the ‘best’ tree topology search operation (42). The tree was visualized with FigTree (http://tree.bio.ed.ac.uk/software/figtree), then edited with Inkscape (http://www.inkscape.org). Accession numbers for human and *A. thaliana* proteins and alignments are presented in Supplementary File S1 and Supplementary Figure S1 respectively.

### Plasmid construction

To determine the localization of TgSR3, we replaced the 3′ region of the gene with three haemagglutinin (HA) tags. We PCR amplified a 2.4 kb fragment from the 3′ end of the TgSR3 gene using genomic DNA as template. This fragment was annealed into the pLIC-HA3/DHFR plasmid (a kind gift from Michael White, University of South Florida) as described previously (43). For the conditional-overexpression mutant, the entire coding sequence of TgSR3 was amplified by PCR from complementary DNA. This fragment was ligated into the pCTDDnH plasmid (44), with PCR product and vector digested with XmaI and AatII. Primers are listed in Supplementary File S2.

### *Toxoplasma gondii* culture and manipulation

*Toxoplasma gondii* parasites were cultured on human foreskin fibroblasts, transfected, then cloned by limiting dilution, as previously described (45), with selection in 1 μM pyrimethamine (46). Δ*ku80*(+hxgprt) tachyzoites were used for 3′-replacement homologous integration as previously described (43), and RH(Δhxgprt) tachyzoites were used for episomal transfection for the conditional mutant. Plaque assays were performed as previously described (47), scanned, then analyzed with FIJI (48). The relative area of plaques were log transformed before statistical analysis with Student's *t*-test.

### Protein analyses

Western blotting of parasite-derived protein was performed as previously described (47). Rat anti-HA primary antibody (Roche, Australia) and anti-rat horseradish peroxidase secondary antibody (Pierce, Australia) were diluted 1/100 and 1/1000 respectively. Mouse anti-GRA8 primary antibody (a kind gift from Gary Ward, University of Vermont; (49)) and anti-mouse horseradish peroxidase secondary antibody (Pierce, Australia) were diluted 1/10 000 and 1/5000, respectively.

### Microscopy

Immunofluorescence assays were performed as previously described (47). Rat anti-HA primary antibody (Roche, Australia) and goat anti-rat secondary antibody conjugated to Alexa Fluor^®^ 488 (Life Technologies, Australia) were diluted to 1/100 and 1/200, respectively. Parasites were incubated in 200 µg/ml Hoechst 33258 to label the nucleus. Images were acquired with a Leica SP2 confocal microscope, adjusted for brightness and contrast, and merged using the bundled software, then assembled into panels using Inkscape (http://www.inkscape.org).

### RNA preparation and manipulation

Intracellular parasites were grown in T175 flasks for 50–53 h, then purified from host cells (45). RNA was extracted using an Isolate II RNA Mini Kit (Bioline, Australia), as per the manufacturer's instructions. For RNA-seq, RNA samples were provided to AGRF (Melbourne) for cDNA library construction and mRNA sequencing (poly-A enrichment) on an Illumina HiSeq 2000.

For qRT-PCR, cDNA was synthesized with a SMARTer^®^ PCR cDNA Synthesis Kit (Clontech, Australia), as per the manufacturer's instructions. qPCR was performed on a StepOnePlus™ (Life Technologies, Australia), using RT^2^; SYBR^®^ Green qPCR Mastermix (Qiagen, Australia), as per the manufacturer's instructions. qRT-PCR experiments were validated using no-RT and no-RNA negative controls. Relative abundance of transcripts was log converted before statistical analysis with Student's *t*-test, and Bonferroni correction was used to adjust for multiple hypothesis testing with *n* = 27.

### Bioinformatic analyses

Analyses were performed on an IBM iDataplex x86 supercomputer (VLSCI, Australia), or personal computers. RNA-seq data were checked for quality with FastQC (http://www.bioinformatics.babraham.ac.uk/projects/fastqc) before mapping with Tophat2 (50). Mapping was checked for quality with flagstats (51) and RNA-SeQC (52). Gene models were created with Cufflinks and models from all samples combined with Cuffmerge (53). Overlapping transcripts were then trimmed to coding-region boundaries with GeneGuillotine, which we have developed and made available as open-source software (available from https://github.com/protist/GeneGuillotine). Mapping and gene models were visualized in IGV (54).

Samples were analyzed for whole-gene differential expression with limma/voom (55), and for differences in alternative-splicing with DEXSeq (56). Statistically-significant events were exported and manipulated, then proportional Venn diagrams created with BioVenn (57). Heat maps were created by importing event values into Gnumeric, sorting and applying conditional formatting to cells (http://www.gnumeric.org). Pathway enrichment was analyzed with GOstat (58), using gene ontology categories extracted from GFF files from ToxoDB (39). The extent of alternative splicing under single condition was quantified using JunctionJuror, which we developed and made open-source at https://github.com/protist/JunctionJuror. Program version numbers and detailed commands are specified in Supplementary File S3.

The software that we have developed should prove useful to scientists. Here is a description of both programs.

GeneGuillotine reads in a GTF file (e.g. from Cuffmerge), and prevents transcripts from overlapping multiple genes, according to a second (reference) GFF. This might be useful if a downstream program (e.g. DEXSeq) requires each transcript to be separate and not overlapping with its neighbours. The position of the split is determined by the genes in the reference GFF. The default is to constrain each transcript to the limits of the CDS. Transcripts that lie wholly within intergenic regions will be kept. The GTF parser is designed to accept Cuffmerge GTF files. Hence, the canonical usage is to use the Tuxedo pipeline (i.e. map reads with Tophat, create gene models with Cufflinks and merge samples with Cuffmerge). The GFF parser is designed for use with GFF files from EuPathDB. It only parses features marked as CDS (and tRNA and rRNA), since UTR information is not available for all genes. The script firstly splits transcripts that overlap multiple genes. An optional flag is available (-m), to only runs this first split. The second part of the script then truncates transcripts that lie on adjacent genes, but overlap with each other. The script outputs a modified GTF file, with gene IDs from the reference gff file written to the transcripts, presuming the transcript overlaps a gene. In other cases, the nearest gene is recorded. For intergenic transcripts, ‘after_GENE_ID’ or ‘before_GENE_ID’; and for transcripts that lie before the first gene or after the last gene, ‘before_first_GENE_ID’ or ‘after_last_GENE_ID’. If there are no genes on the reference contig, the gene ID is ‘No_genes_on_ref_contig’. If transcripts cover multiple genes, then the transcripts will be renamed to ‘TRANSCRIPT_ID’, ‘TRANSCRIPT_ID:2’, ‘TRANSCRIPT_ID:3’, etc. These strings can be easily modified from the code.

JunctionJuror will identify the amount of alternative splicing arising from differential splice-site usage, given a junction.bed file and .gff genome file (i.e. it ignores intron retention). This script accepts junction.bed files, which should be referenced in a space-delimited list, with each line comprising a path to a junction.bed file, followed by a condition. The included test files show an example of the format. The current version of JunctionJuror analyses single conditions, and hence this file should only contain reference to one condition. The GFF parser is also designed for use with GFF files from EuPathDB. Similarly to GeneGuillotine, it only parses features marked as CDS (and tRNA and rRNA). JunctionJuror outputs the list of genes that are considered to be confirmed as alternatively spliced. The threshold for confirmation can be specified by users. If a particular junction is present in (at least) this number of replicates, JunctionJuror will accept this junction as confirmed. This threshold defaults to two. Finally, instead of reporting alternatively-spliced genes, an optional flag makes JunctionJuror list multi-exon genes, i.e. genes containing at least one confirmed junction, as specified by the threshold.

## RESULTS

### Bioinformatic identification of putative SR proteins in *T. gondii* and *P. falciparum*

**Figure 1.**
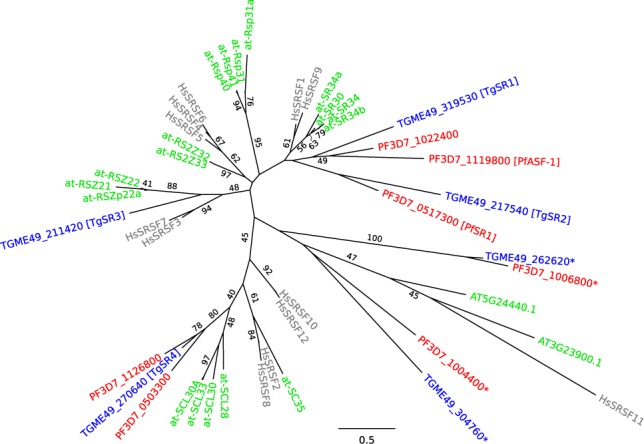
Maximum-likelihood phylogenetic tree for SR proteins. Grey text (starting with ‘Hs’) represents human proteins, green text represents *Arabidopsis thaliana* proteins (those starting with ‘at-’ have been previously identified as SR proteins (60)), blue text (starting with ‘TG’) represents *T. gondii* genes, and red text (starting with ‘PF’) represents *P. falciparum* genes. The asterisked genes were considered too divergent for further investigation. Maximum-likelihood bootstrap values for 100 replicates are only shown on branches if they are 40 or above. The scale bar indicates 0.5 substitutions per amino acid.

In humans, there are 12 known SR proteins, SRSF1–SRSF12 (34,35). We generated an alignment of these proteins with *A. thaliana*, *T. gondii* and *P. falciparum* homologues, and inferred a phylogenetic tree from this alignment (Figure 1). Given the short region of conservation in the SR proteins, in the N-terminal RNA-recognition motif, bootstrap values were all fairly low.

There was no clear one-to-one orthology between any of the apicomplexan and human genes, nor between the apicomplexan and plant genes. In particular, the most well-studied alternative-splicing SR protein, SRSF1 (also known as ASF or alternative-splicing factor), had no clear apicomplexan orthologue.

SRSF1 was placed in an unresolved clade with two *T. gondii* genes, which we dubbed TgSR1 and TgSR2, and three *P. falciparum* genes, including the previously-identified PfASF-1 (59) and PfSR1 (29). This clade also appeared to include members from the *A thaliana* SR subfamily (60), although bootstrap values were too low for any confidence.

One apicomplexan clade lay on a relatively long branch (PF3D7_1006800 and TGME49_262620; asterisked in Figure 1). These were potentially too divergent from canonical SR proteins, and hence were excluded from further analysis. Similarly, two other apicomplexan proteins (PF3D7_1004400 and TGME49_304760; also asterisked) clustered adjacently with long branches, and were also removed. These appeared in the same clade as two other *A. thaliana* proteins (AT3G23900.1 and AT5G24440.1), which had not previously been identified as SR proteins (60).

The two remaining *T. gondii* genes were named TgSR3 and TgSR4. As there was no clear one-to-one orthology with existing genes, our numbering bears no relationship to the numbering of the human orthologues. Of the four *T. gondii* homologues identified, TgSR3 had the highest reported expression levels (39). We selected this homologue for further investigation, including conditional overexpression, as it would most likely give the most robust and observable effects (see below). TgSR3 appeared to fall within a clade that included HsSRSF3, HsSRSF7 and the *A thaliana* RSZ subfamily (60), although the bootstrap value of 48% was too low for confidence.

### TgSR3 localizes to nuclear-speckle–like structures in *T. gondii*

**Figure 2.**
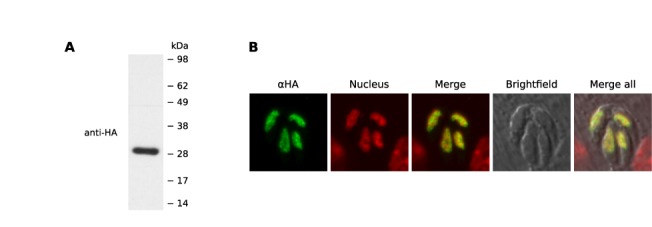
(**A**) Western blot of total protein from purified 3′-replacement TgSR3-HA parasites. (**B**) Confocal-microscopy maximum projections of immunofluorescence assays for the 3′-replacement transfectants. The green channel represents localization of HA-tagged TgSR3 protein. The red channel represents the nucleus, as stained by Hoechst. The merge of these two columns is shown in the third panel, the brightfield image follows, and the final panel is the merge of the other four.

To determine the localization of the *T. gondii* homologue, TgSR3, we introduced three HA tags at the 3′end of the endogenous gene using single-site recombination in *Δku80* strain parasites (43). Monoclonal transfectants were obtained, and total protein from purified intracellular tachyzoites was analyzed by western blot. Probing with an anti-HA antibody revealed a protein around 29 kDa, similar to the predicted mass of 25 kDa (Figure 2A).

Immunofluorescence assay (IFA) of the tagged TgSR3 showed the protein localizing in a speckle-like pattern in the nucleus (Figure 2B). TgSR3 is absent from a region in the nucleus that lacks DNA, likely corresponding to the nucleous. This nuclear localization and exclusion from the nucleolus is consistent with the nuclear-speckle localization of human SR proteins and *P. falciparum* PfSR1, although PfSR1 was sometimes non-nuclear in different erythrocytic stages (61).

### Conditional perturbation of a putative alternative splicing factor

We created conditional overexpression mutants for TgSR3 using the previously-described ddFKBP system (62). We created a construct consisting of an N-terminal FKBP destabilizing domain (DD), followed by an HA epitope and the full-length protein-coding cDNA for TgSR3. This integrated randomly into the *T. gondii* genome, creating an additional copy of TgSR3. Normally, proteins linked to a DD are degraded quickly by the proteasome (63). In the presence of Shld1, DD-tagged proteins are rapidly protected from this degradation, resulting in net overexpression of TgSR3. This rapid induction of overexpression allowed us to compare uninduced and induced lines, without fear of deleterious effects or secondary adaptation to long-term overexpression. In addition, the expression of DD-tagged proteins is tuneable in a dose-dependent manner. One drawback of the DD system is that it is slightly ‘leaky’ in the absence of Shld1, with small amounts of protein not degraded. However, by selecting the more highly-expressed SR homologue, this leakiness would have a proportionally minor effect compared to the high endogenous expression.

**Figure 3.**
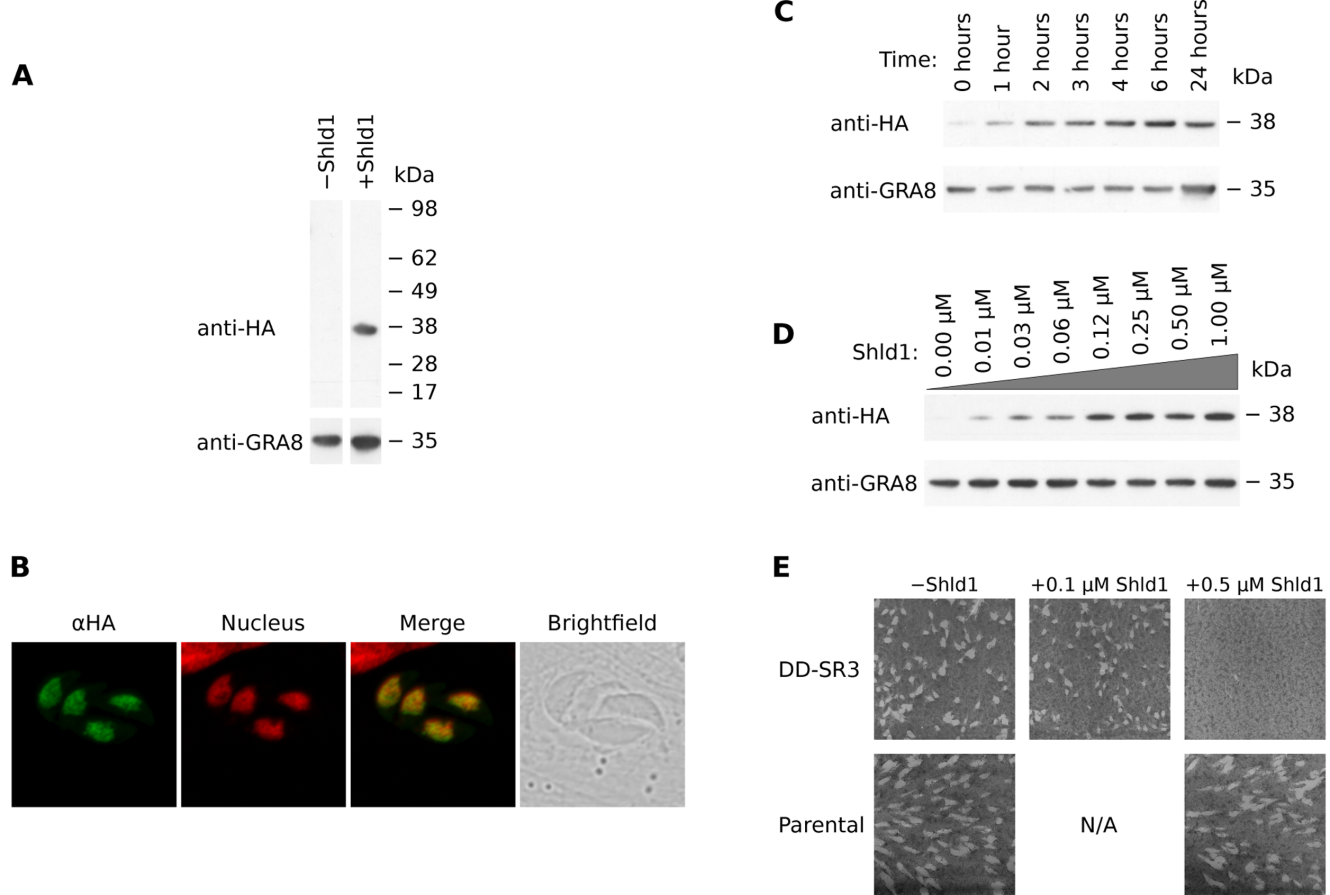
Assays performed on conditional mutants for TgSR3 on a range of Shld1 conditions. (**A**) Western blot of total protein from purified parasites in the presence and absence of Shld1. Anti-HA was used to detect tagged TgSR3 protein and anti-GRA8 was used as a loading control. Each row is taken from the same blot. (**B**) Confocal microscopy maximum projections of immunofluorescence assays for mutants in the presence of Shld1. The green channel represents localization of HA-tagged SR protein. The red channel represents the nucleus, as stained by Hoechst. The third pane shows the merge of these two panes, and the brightfield image is last. (**C**) Western blot of total protein from purified parasites for different durations of incubation with 1 µM Shld1. (**D**) Western blot of total protein from purified parasites with different concentrations of Shld1 added for 24 h. (**E**) Plaque assays for conditional mutant and parental parasites. Compared to uninduced transfectants, the plaques of induced transfectants were slightly but significantly reduced in size at 0.5 µM (22% reduction in area; *P* value = 3.2 × 10^−7^). At 0.5 and 1 µM (latter not shown), no plaques were detected. Plaque size of parental parasites were unaffected when 0.5 µM Shld1 was added.

Monoclonal transfectants were created, and analyzed by western blot and immunofluorescence assay in the absence and presence of 1 µM Shld1; this concentration was previously reported to have no effect on the growth of wild-type parasites (64). In the presence of Shld1, tagged TgSR3 was detected by western blot. In the absence of Shld1, no tagged TgSR3 was detected (Figure 3A). The observed mass of 35 kDa was consistent with the predicted mass of 34.6 kDa.

We also confirmed that nuclear targeting of this N-terminal DD-HA-tagged TgSR3 was unaffected. After adding Shld1, localization 24 h later by IFA was consistent with our 3′-replacement results above, with a subnuclear, speckle-like distribution that excludes the nucleolus (Figure 3B).

The conditional mutant yielded different concentrations of tagged TgSR3, dependent on both duration and amount of Shld1 added. We added 1 µM Shld1 to parasites for different durations, harvested total protein from purified parasites, then assayed by western blot (Figure 3C). The amount of tagged TgSR3 increased gradually from 0 h, before stabilizing around 4 h after addition of Shld1. We also incubated with Shld1 for 24 h over a range on concentrations, before assaying by western blot (Figure 3D). The concentration of tagged TgSR3 increased with the concentration of Shld1, before levelling out around 0.1 µM Shld1.

### Overexpression of TgSR3 causes a growth defect

To determine if overexpression of TgSR3 would result in a growth defect, we performed a series of plaque assays. Flasks of the conditional mutant were incubated in the presence of either 0, 0.1, 0.5 or 1 µM Shld1 for nine days (Figure 3E). Parasites undergo multiple lytic cycles, clearing regions of the host cell monolayer. The size of individual plaques represents the amount of replication and growth that has occurred. Adding 0.1 µM Shld1 resulted in a slight but significant reduction in plaque size compared to untreated parasites (22% reduction in plaque area; *P* value =3.2 × 10^−7^, Student's *t*-test), indicating a defect in growth. Adding 0.5 or 1 µM Shld1 produced no visible plaques, consistent with few or no completed lytic cycles. This suggests that overexpression of the TgSR3 SR protein homologue causes a severe deficiency in growth.

We also assayed parental parasites, to quantify any negative reaction to Shld1. 1 µM Shld1 was previously reported to not affect growth of wild-type parasites (64). In the presence of 0.5 µM Shld1, plaque sizes were not significantly different to untreated parasites, implying that the growth defect in the mutant was purely due to overexpression of TgSR3. Under our conditions, adding 1 µM Shld1 resulted in a significant growth defect (38% reduction in plaque area; *P* value = 1.2 × 10^−5^; data not shown). Hence, for subsequent experiments, a maximum of 0.5 µM Shld1 was added to parasites.

### TgSR3 perturbs alternative splicing

**Figure 4.**
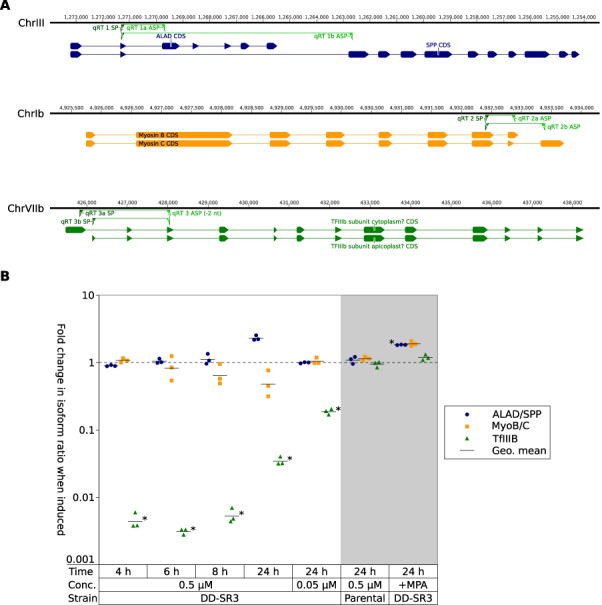
qRT-PCR of three known alternatively-spliced genes. (**A**) Schematic showing coding sequence of isoforms tested. Numbers on horizontal axis indicate position in the chromosome, as per ToxoDB (39). Solid blocks indicate exons, and joining lines indicate introns (untranslated regions not shown). Green triangles show primer-binding sites (the 3’ end of qRT 3 ASP binds on the other side of the intron). (**B**) Change in isoform ratio between isoform A and B ($\Delta \mathrm{isoform\_ratio}$) for these three genes, for different conditions as per the x-axis. The unshaded area represents addition of 0.5 µM Shld1 to the conditional mutant for 4, 6, 8 or 24 h; and addition of 0.05 µM Shld1 for 24 h. The shaded area represents controls: the parental strain with 0.5 µM Shld1 added for 24 h; and treatment of the mutant with 25 µg/ml mycophenolic acid (MPA) for 24 h. Horizontal lines show the geometric mean of triplicates. * indicates statistical significance with adjusted *P* value <0.05.

To determine the cause of the growth defect, we wanted to confirm TgSR3's putative role as an alternative-splicing factor. We used quantitative reverse-transcription polymerase-chain reaction (qRT-PCR) on three known alternatively-spliced genes to see if the ratios of their alternatively-spliced isoforms would change with overexpression of TgSR3. The first gene analyzed encodes both delta-aminolevulinic acid dehydratase (ALAD; also known as porphobilinogen synthase; TGGT1_253900) and the stromal processing peptidase (SPP; TGGT1_253890). These genes have independent functions; the former is necessary for haem synthesis, whereas the latter cleaves apicoplast-targeting leaders. In *P. falciparum*, both enzymes localize to the apicoplast, via a shared leader that is alternatively-spliced (9,65) (Figure 4A). This splicing is conserved in *T. gondii* (66).

The second gene chosen was myosin B/C (TGGT1_255190). The myosin B/C gene produces two splice forms that differ in their C-termini (Figure 4A), which confers differences in solubility and localization (10).

The final gene was the transcription factor IIIb subunit (TFIIIB; TGGT1_207900). This gene has previously been reported as being alternatively spliced (67), with a shorter form translated from an alternative downstream start codon according to RNA-seq data (39), and hence alternatively spliced to the second exon (Figure 4A). Analysis with SignalP and ApicoAP predicted a signal peptide and apicoplast leader in this minor isoform, with no signal peptide detected in the canonical isoform (68,69). This is an example of alternative splicing of the 5′ splice site of an intron.

We designed primers to distinguish between the two primary alternatively-spliced forms for each of the three genes (Figure 4A), and then created cDNA samples for different conditions. We extracted samples from our conditional mutant in tachyzoites in the absence of Shld1, and after incubation with 0.5 µM Shld1 for 4, 6, 8 or 24 h. We also generated a sample incubated for 24 h with only 0.05 µM Shld1. For each of these induced samples, we quantified the amount of mRNA for each isoform using qRT-PCR, then expressed this as a ratio between isoforms *within* each sample condition (i.e. $\mathrm{isoform\_ratio}=\left[\mathrm{isoform\:1a}\right]/\left[\mathrm{isoform\:1b}\right]$). We then determined how much this isoform ratio had changed after induction, by comparing ratios of treated samples with untreated samples (i.e. $\Delta \mathrm{isoform\_ratio}=\mathrm{isoform\_ratio_{induced}}/\mathrm{isoform\_ratio_{uninduced}}$). This value is shown in Figure 4B (unshaded region), for the three genes in technical triplicates. If alternative splicing of a gene is not perturbed when TgSR3 is overexpressed, we expect this $\Delta \mathrm{isoform\_ratio}$ to not significantly differ from 1. This is the case for ALAD/SPP and myosin B/C, which implies that their alternative splicing is not regulated by TgSR3.

In comparison, the $\Delta \mathrm{isoform\_ratio}$ for TFIIIB was significantly different from 1, suggesting that its alternative splicing is perturbed when TgSR3 is overexpressed. This change in isoform ratio ($\Delta \mathrm{isoform\_ratio}$) was most pronounced at the 6-h time point (adjusted *P* value 6.9 × 10^−7^). When induced, 220-fold more of the shorter, apicoplast-predicted isoform was detected when normalized to the canonical isoform. Alternative splicing of TFIIIB was also perturbed at other time points, with the extent of perturbation decreasing to 24 h. As expected, the ratio was also less pronounced when incubated with the lower concentration of 0.05 µM Shld1.

As a control, the $\Delta \mathrm{isoform\_ratio}$ was calculated for parental parasites, after incubation with 0.5 µM Shld1 for 24 h (Figure 4B, shaded region). The isoform ratio did not significantly change for any of the three genes, validating that the perturbation of TFIIIB's alternative splicing was indeed caused by overexpression of TgSR3. As an additional control, we incubated mutant parasites for 24 h with 25 µg/ml mycophenolic acid, which is a lethal concentration of this drug for parasites lacking HXGPRT (46). There was no significant change in isoform ratio for TFIIIB, which suggests that our reported change in alternative splicing directly arises from the overexpression of TgSR3, rather than being a downstream response to general parasite death.

### Deep sequencing shows differential expression of genes when overexpressing TgSR3

In order to determine how much alternative splicing is modulated by TgSR3 globally, we used RNA-seq to sequence the mRNA transcriptome of our mutant. We sequenced cDNA from the conditional mutant in the absence of Shld1, and after incubation with 0.5 µM Shld1 for 4, 8 or 24 h, in biological triplicates. The 12 samples were sequenced on one lane of an Illumina HiSeq, resulting in an average of 15.9 million paired reads (2 × 100 bp) per sample. These reads were mapped to the *T. gondii* genome, then gene models constructed.

**Figure 5.**
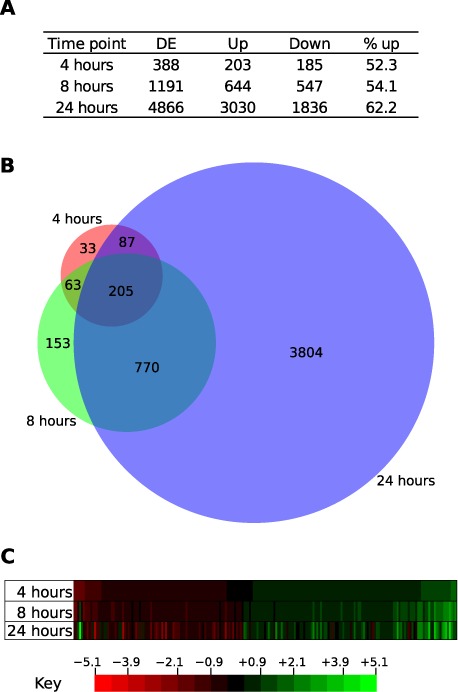
Differential expression analysis of whole genes. (**A**) Number of genes differentially expressed (DE), and specifically upregulated (up) or downregulated (down) when Shld1 was added for three different durations. (**B**) Proportional Venn diagram showing the commonality of genes at each time point. (**C**) Heatmap of fold change in relative abundance for genes significantly changed at all time points. Bins are ordered by fold change at the 4-h time point.

First, we investigated whether overall expression of whole genes was affected by overexpression of TgSR3. Unsurprisingly, the number of genes differentially expressed increased with the length of treatment with Shld1 (Figure 5A). At 4 h, ∼5% of the genome was differentially expressed, increasing to over half of the genome at 24 h.

There were slightly more genes with overall expression upregulated than downregulated, with around 52–62% of differentially-expressed genes upregulated at different time points. There was no clear pathway enrichment detected in these genes, either by analysis of each time point individually, or by intersections, unions, set differences or permutations of these, based on GOstat analysis (58). The vast majority of the differentially-expressed genes were unique to the 24-h time point (74%) (Figure 5B). The full lists of differentially-expressed genes after 4, 8 or 24 h are presented in Supplementary Files S4–S6, respectively.

In order to analyze the level of concordance in fold change between time points, we visualized differential expression with a heatmap. We analyzed the intersection of the sets above, which corresponds to the genes where expression significantly changes at all time points, compared to uninduced transfectants. Genes were ordered by magnitude of fold-change at the 4-h time point (Figure 5C). For each gene, there appears to be a strong correlation in magnitude between all time points.

### TgSR3 perturbs alternative splicing in many genes

**Figure 6.**
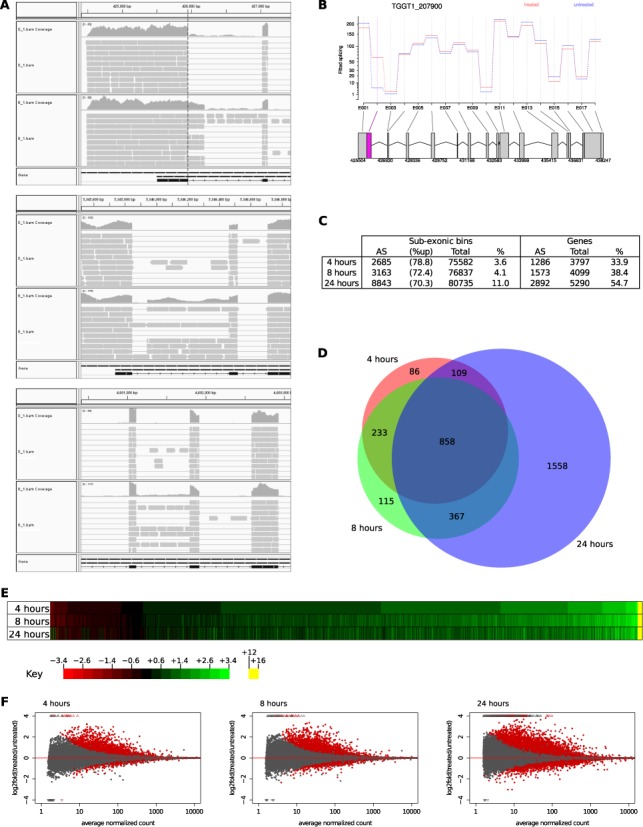
RNA-seq analysis of exon-level differential expression of the conditional-expression mutant. (**A**) A schematic of RNA-seq reads (grey boxes) mapping to the genome at the start of TFIIIB. The large top pane (‘0_1’) indicates uninduced parasites, and the second large pane (‘8_1’) was treatment with Shld1 for 8 h. The ‘coverage’ graphs indicate the number of reads mapping to each base position. The narrow lowest pane (‘Gene’) shows the canonical gene model from ToxoDB. Top panel: alternative 5′ splice-site, in the 5′ end of TFIIIB. Compared to the uninduced sample (‘0_1’), more reads mapped in the induced sample (‘8_1’) to an intron of the canonical gene model, shown to the right of the vertical dashed line. Middle panel: alternative 3′ splice-site, in TGGT1_270770. Bottom panel: intron retention, in TGGT1_271350. (Images originally created with IGV (54).) (**B**) DEXSeq is able to detect changes in alternative splicing of TFIIIB. The gene model at the bottom defines potential sub-exonic bins (boxes). The graph indicates number of reads from the uninduced sample (blue) and after 8-h incubation with Shld1 (red). Here, the numbers of reads differ in the second sub-exonic bin, with proportionally more reads mapping in the treated samples. Statistical significance is indicated by the magenta exon. (Image originally created with DEXSeq (56).) (**C**) Table showing the number of sub-exonic bins differentially expressed (with % of these that were upregulated), and the resulting number of genes in which alternative splicing was perturbed. This is then expressed as a percentage (%) of alternative-splicing (AS) divided by either the total number of bins delineated or genes where expression was detected (total). (**D**) Proportional Venn diagram showing the commonality of genes affected at each time point. (**E**) Heatmap of fold change for bins significantly changed at all time points. Bins are ordered by fold change at the 4-h time point. (**F**) MA plots for each time point, where M (y-axis) represents the log_2_-fold ratios of counts in treated bins over counts in untreated bins, and A (x-axis) represents the average normalized count within bins. Hence, the position on the y-axis shows the amount of differential expression, with greater than 0 indicating an increase in proportion when Shld1 is added. The position on the x-axis represents the number of reads within each bin. Each dot represents a bin, with red indicating statistical significance of differential expression (adjusted *P* value < 0.05).

The primary purpose in generating RNA-seq data was to determine how much alternative splicing was modulated globally by TgSR3. As a proof of concept, we first manually confirmed that the data could reveal changes in alternative splicing for TFIIIB. Under normal conditions, RNA-seq data confirm the position of the first intron (Figure 6A, top panel, ‘0_1’, splice site at the vertical dashed line). When TgSR3 is overexpressed (Figure 6A, top panel, ‘8_1’), we see proportionally more reads mapping within this intron of the canonical gene model (to the right of the vertical dashed line). This indicates an increase in the proportion of the shorter isoform, consistent with our qRT-PCR results.

In addition to alternative 5′ splice sites, we also saw other classes of alternative splicing, which were later confirmed to be statistically significant by downstream analyses (see below). This included alternative 3′ splice sites (Figure 6A, middle panel), and intron retention (Figure 6A, bottom panel).

To screen all genes for perturbation of alternative splicing, a program called DEXSeq was used (56). This program divides each gene at potential alternative-splice-site boundaries, then counts the number of reads that fall into each bin. It then compares different samples, to see if the number of reads within each sub-exonic bin changes. If the bins are differentially expressed, this implies that overexpression of TgSR3 has induced a change in the alternative-splicing topology for this gene.

DEXSeq normalizes each gene to the total number of reads within the transcripts, and hence requires accurate transcript models to function correctly. In parasites such as *T. gondii*, the compact genome often results in overlapping transcripts, both sense and anti-sense to each other. Non-overlapping transcripts must be obtained for downstream analysis with DEXSeq. We developed a new methodology, creating a program called GeneGuillotine to restrict transcript isoforms to CDS regions as defined by gene models, in our case from ToxoDB (39). If transcript ends extend into untranslated regions, they will be truncated by GeneGuillotine. If they are are wholly intergenic, then they are presumed to be novel, and untouched. This program is open-source and freely available from https://github.com/protist/GeneGuillotine.

We confirmed the efficacy of DEXSeq by manual inspection of the TFIIIB locus, where DEXSeq was able to detect a change in alternative splicing (Figure 6B, magenta box). Overexpression of TgSR3 results in proportionally more of the second sub-exonic bin, and hence a perturbation of alternative splicing to increase the amount of the shorter isoform. This again confirms the qRT-PCR results.

Once this pipeline was confirmed to work for a single gene, we extended the screen to the entire transcriptome. As expected, the number of differentially-expressed sub-exonic bins increased with time of induction (Figure 6C, sub-exonic bins, %). Similarly, the number of genes where alternative splicing was perturbed increased with time (Figure 6C, genes, %). In both cases, there was a considerable jump at the 24-h time point, possibly from pleiotropic events.

Again, we visualized the overlap at different time points for these affected genes (Figure 6D). Similar to the whole-gene differential-expression analysis, this showed a large proportion of genes unique to the 24-h time point, although this dropped to 47% of affected genes. The full lists of genes with differentially-expressed exonic bins after 4, 8 or 24 h are presented in Supplementary Files S7–S9 respectively.

Similarly, we compared fold change between time points with a heatmap. Using the intersection of the sets above, bins were again ordered by magnitude of fold-change at the 4-h time point (Figure 6E). With the increased data points, there appears to be a very strong correlation for all time points.

The alternatively-spliced genes perturbed by TgSR3 were analyzed for pathway enrichment. We saw similar results whether we analyzed each time point individually, or the intersection of all these sets. In the latter case, we saw an over-representation of genes involved in ATPase activity (adjusted *P* value = 0.0013), and purine ribonucleotide binding (adjusted *P* value = 0.0083).

When TgSR3 is overexpressed, 70.3–78.8% of changes to alternative splicing arise from upregulation of bins (Figure 6C). This can be visualized graphically with MA plots (Figure 6F). These graphs depict M (log_2_-fold ratios of counts in treated bins over counts in untreated bins) on the y-axis and A (average normalized count within bins) on the x-axis, and also show the relationship between these parameters and statistical significance (red dots in the Figure). These plots clearly show that there are more significantly-perturbed bins with a positive fold-change. This bias towards upregulation is not only seen in the number of bins affected, but also the extent of this fold change. For example, at the 8-h time point, the change in expression of sub-exonic bins has a maximal reduction of 5.1-fold, but a maximal increase of 42 000-fold.

### There are over 1900 alternatively spliced genes in *T. gondii*

In order to quantify the number of genes that are alternatively spliced in a single condition, we wrote another program for this analysis, which we named JunctionJuror. This program assigns junction reads observed in all three replicates to specific genes, before detecting overlapping junctions, which indicate alternative splicing. That is, reads bridging across a specific region indicate the possible presence of an intron. If a junction is observed in all three replicates, it is considered to be high confidence, and will be analyzed downstream. After high-confidence reads have been collated, JunctionJuror then looks for discordant junctions that overlap with each other; the presence of mutually exclusive introns indicates an alternative splicing event.

Hence, JunctionJuror is capable of detecting most forms of alternative splicing, including exon skipping, mutually exclusive exons, and alternative 5′ and 3′ splice sites, with the exception of intron retention. While intron retention is potentially an important type of alternative splicing, we decided to overlook this subset due to the availability of junction reads as a high-confidence indicator of alternative splicing. This program is open-source and freely available from https://github.com/protist/JunctionJuror.

Using JunctionJuror, we calculated the number of alternatively-spliced genes in our samples, ignoring intron retention. For uninduced samples, we detected alternative splicing in 1914 genes. At 4 h, this number slightly decreased to 1891 genes, before rising again at 8 and 24 h to 2214 and 2700 genes respectively. These represent 22.6%, 22.3%, 26.2% and 31.9% respectively of all annotated tRNA and protein-coding genes. The lists of alternatively-spliced genes in uninduced samples, and after induction for 4, 8 or 24 h are contained in Supplementary Files S10–S13.

Despite the lack of detection of intron retention, these genes that were identified as being alternatively spliced in single conditions still correlated well with genes identified in the previous section, with their alternative splicing perturbed by TgSR3. For example, 82% of genes that had their alternative splicing perturbed by overexpression of TgSR3 after 8 h were also identified as being constitutively alternatively spliced in uninduced and/or 4-h induced samples. Visual analyses are available in Supplementary Figure S2. We also attempted pathway enrichment analysis, which revealed no enriched functions.

## DISCUSSION

A number of analyses of individual spliced transcripts underline the biological importance of alternative splicing in apicomplexan parasites. In *P. falciparum* and *T. gondii*, alternative splicing is involved in providing correct targeting sequences to adjacent genes (9), allowing multiple localizations of single genes (7,8,10–12), and generating diversity of surface proteins (13,14). These studies show that alternative splicing is often a regulated process that produces biologically-significant protein diversity, necessary for parasite survival and proliferation.

Despite the importance of alternative splicing, few deep-sequencing experiments have explicitly addressed its extent in *Plasmodium* or *Toxoplasma*. Nevertheless, RNA-seq experiments designed to improve annotation of gene models or to assemble whole transcriptomes (and which use algorithms not explicitly suited to detection of alternative splicing) have revealed that many *Plasmodium* and *Toxoplasma* genes are indeed alternatively spliced, with alternative splicing detected for ∼5% of genes analyzed (31–33). However, these analyses use cDNA derived from a single life stage, either the intraerythrocytic stage of *P. falciparum*, or *T. gondii* from mouse bone-marrow macrophages. In addition, deficiencies inherent in previously-employed bioinformatic analyses that were not explicitly designed to detect novel splice variants can significantly underestimate the true rate of alternative splicing (56). In comparison, a targeted approach found 16% of analyzed *Plasmodium* genes were alternatively spliced (30), although this suffered from limited depth of sequencing and was again restricted to a single stage. Further, limited analysis of RNA-seq experiments for a few *P. falciparum* stages indicated widespread differences in alternative splicing (70). These data indicate that alternative splicing is widespread in apicomplexan genomes and generates diversity in transcripts between life stages.

Although RNA-seq can be effective in detecting alternative splicing in single samples, the level of detection is limited by the depth of sequencing (33). We have identified and overexpressed an SR protein, TgSR3, which causes perturbation of alternative splicing. The perturbation of TgSR3 allows detection of alternative-splicing events dependent on this alternative-splicing factor. To our knowledge, this is the first time that overexpressing SR-protein mutants have been analyzed with RNA-seq, and is a novel application of both technologies.

Our conditional mutant showed that overexpression of TgSR3 is deleterious to the parasite, presumably from large-scale perturbation of the transcriptome. From our qRT-PCR data, alternative splicing of transcription factor IIIb subunit (TFIIIB) was perturbed most at the 6-h time point, before decreasing at 24 h post-induction (Figure 4B). This could potentially be due to pleiotropic effects from parasites responding to the TgSR3 overexpression. Hence, we considered splice changes seen only in the 24-h-post-induction condition likely to represent mainly secondary effects. This interpretation is supported by analysis of RNA-seq data below.

Analysis of RNA-seq data from pathogens poses novel challenges, demanding new bioinformatic techniques and tools. Pathogens have compact genomes, and transcripts from adjacent genes often overlap. Transcripts must be unconnected to prevent computational issues in downstream analyses. This is particularly relevant when screening for alternative splicing, where read depth within bins should be normalized to whole-gene expression. We trialled several existing computational pipelines to screen for alternative splicing. Many of these, such as CuffDiff, compare expression ratios of full-length transcript isoforms (71). In comparison, more statistical power can be gained by exon-level analysis of alternative splicing (56,72). Further, only a few tools use this approach with multiple replicates; one of these is DEXSeq (56). DEXSeq works well as a screen, but does not utilize information from bridging RNA-seq reads, and thus cannot reliably deduce splice-junction locations. We are aware of only a few tools at present that satisfy all of these requirements. We attempted to use MATS (73), but the software failed to work with our data, and the authors were unable to make it functional. Similarly, DiffSplice was unable to identify known splicing events in our data (74).

In order for the DEXseq pipeline to function correctly for compact genomes, we had to develop a new bioinformatic tool, which we have named GeneGuillotine. We trialled different methods of splitting transcripts on adjacent genes, including splitting at the minimum read depth. However, we found that this resulted in numerous false positives downstream. The most reliable method was to be conservative, and restrict transcript boundaries to the extremities of the proposed coding sequence from existing gene models. Hence, when given existing gene models, GeneGuillotine is capable of coalescing RNA-seq transcript reads into distinct genes, removing any overlap arising from compact genomes. GeneGuillotine has been released to the public as free and open-source software available at https://github.com/protist/GeneGuillotine. This provides a useful resource for scientists that wish to normalize RNA-seq data to individual genes. Further, GeneGuillotine will be useful to a wide variety of RNA-seq applications where separation of adjacent transcripts are required.

RNA-seq analysis suggests that overexpression of TgSR3 specifically affects alternative splicing, with expression of whole genes perturbed as a downstream consequence. This is supported by comparison of whole-gene differential expression with alternative-splicing analysis. First, the number of whole genes differentially expressed increases from 388 genes to 4866 genes at the 4 and 24 h time points respectively, a 13-fold increase. In comparison, the amount of genes with alternative splicing perturbed increases from 1286 to 2892, a 2-fold increase. Concomitant with this is the number of affected genes that overlap at different time points. For whole-gene differential expression, only 4.0% of the perturbed genes are shared by all time points, with 74% unique to the 24-h time point. For perturbation of alternative splicing, these values are 26% and 47%. Thus, immediately after overexpression of TgSR3, alternative splicing for a moderate number of genes is perturbed; as overexpression continues, only a few more genes are affected, with most of the affected genes and bins staying constant. This is supported by the Venn diagram and heatmap. In contrast, the number of whole genes that alter their expression is low initially, before rising rapidly; there are many genes unique to each time point. These data are consistent with the expectation that initially, overexpression of TgSR3 primarily affects alternative splicing of many genes, before downstream stimulation of whole-gene expression changes in a plethora of genes. However, we cannot exclude that overexpressed TgSR3 binds to non-canonical splice sites, including some which may normally be bound by other SR proteins.

In addition, perturbation of alternative splicing appears to occur in a very specific manner. Whilst the number of genes alternatively spliced is relatively high (e.g. 38% at 8 h), the number of bins differentially expressed is quite low (e.g. 4%). This suggests that while many genes are affected by overexpression of TgSR3, only a small, and presumably specific, subset of splicing events are altered within each gene. Similarly, pathway analysis showed enrichment of genes involved in ATPase activity, which is fairly broad, and purine ribonucleotide binding, which potentially results in a transcription-regulatory feedback loop. However, the lack of other pathways identified may imply that TgSR3 is a universal regulator of splicing, fundamental to proper function of alternative splicing in general. Similarly, there was no pattern observed in pathways for alternatively-spliced genes in single samples.

Treated samples exhibited a tendency to upregulation of bins. This represents either inclusion of cryptic exons (intron retention), or elongation of existing exons. The ratio of upregulated bins to downregulated bins drops with length of induction. This implies that overexpression of TgSRm3 initially induces primarily upregulation of bins, with pleiotropic effects at later time points resulting in less-specific perturbation of alternative splicing.

Finally, we attempted to determine the extent of alternative splicing in individual samples. Again, there were no existing programs that suited our needs. Some programs, such as ALEXA-Seq, SpliceTrap and SpliceSeq, are dependent on curated datasets specific to the human genome (75–77), while others, such as SOLAS and MISO, require transcript models to function (78,79). By creating a program that focused purely on junctions, without requiring reconstituted transcripts, we could directly detect the presence of alternative splicing without requiring accurate gene models. We developed a program named JunctionJuror, which is freely available from https://github.com/protist/JunctionJuror. This program detects genes that contain overlapping junctions, which can identify all forms of alternative splicing with the exception of intron retention.

Using JunctionJuror, we identified alternative splicing in 1914 genes in the uninduced sample, and a general increase when induced, up to a maximum of 2700 genes when induced for 24 h. When uninduced, this is equivalent to 22.6% of tRNA and protein coding genes, which is in stark contrast to the 5% reported in previous non-targeted transcriptomic experiments, which focused on reconstituting transcript models (31–33), and more than the 16% reported by targeted small-scale analyses in *Plasmodium* (30). Further, detection of alternative splicing is heavily dependent on sequencing depth; given our sequencing conditions, our experiments have likely captured much less than half of all junctions (78), and hence we would expect the full diversity of alternative splicing to be much greater than this.

## ACCESSION NUMBER

http://www.ncbi.nlm.nih.gov/bioproject/PRJNA252680.

## SUPPLEMENTARY DATA

Supplementary Data are available at NAR Online.

SUPPLEMENTARY DATA
