# Supplementary material for: A serine–arginine-rich (SR) splicing factor modulates alternative splicing of over a thousand genes in Toxoplasma gondii
Source: Nucleic Acids Res. 2015 Apr 13;43(9):4661–75. doi: 10.1093/nar/gkv311 (PMC4482073; doi:10.1093/nar/gkv311)
Supplement: SUPPLEMENTARY DATA [file supp_43_9_4661__index.html]

A serine–arginine-rich (SR) splicing factor modulates alternative splicing of over a thousand genes in Toxoplasma gondii — SUPPLEMENTARY DATA 

# A serine–arginine-rich (SR) splicing factor modulates alternative splicing of over a thousand genes in *Toxoplasma gondii*

## SUPPLEMENTARY DATA

**Files in this Data Supplement:**

- SUPPLEMENTARY DATA
- SUPPLEMENTARY DATA
- SUPPLEMENTARY DATA
- SUPPLEMENTARY DATA
- SUPPLEMENTARY DATA
- SUPPLEMENTARY DATA
- SUPPLEMENTARY DATA
- SUPPLEMENTARY DATA
- SUPPLEMENTARY DATA
- SUPPLEMENTARY DATA
- SUPPLEMENTARY DATA
- SUPPLEMENTARY DATA
- SUPPLEMENTARY DATA
- SUPPLEMENTARY DATA
- SUPPLEMENTARY DATA
