# Supplementary material for: A serine–arginine-rich (SR) splicing factor modulates alternative splicing of over a thousand genes in Toxoplasma gondii
Source: Nucleic Acids Res. 2015 Apr 13;43(9):4661–75. doi: 10.1093/nar/gkv311 (PMC4482073; doi:10.1093/nar/gkv311)
Supplement: SUPPLEMENTARY DATA [file supp_gkv311_nar-03398-a-2014-File010.pdf]

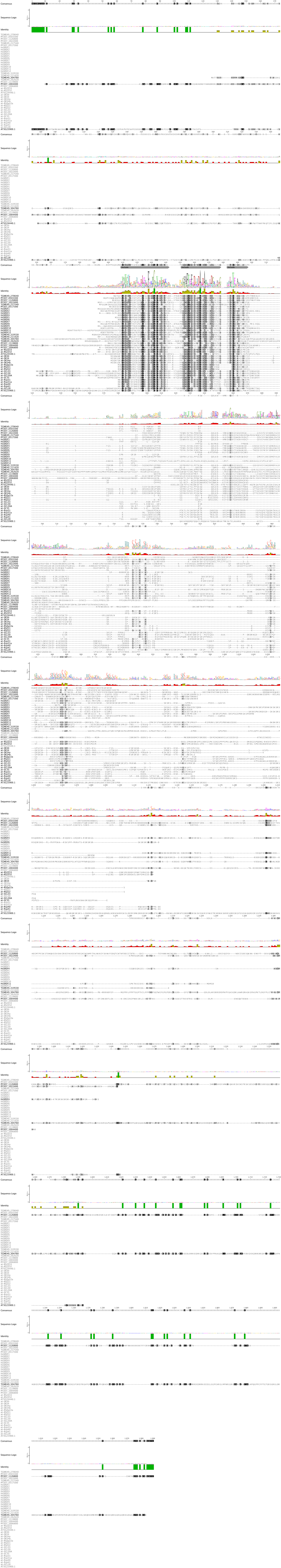
Consensus
Sequence Logo
Identity

Supplementary Figure S1: Alignment used to generate phylogeny of SR proteins. Shading indicates (in order from darkest to lightest), similarity of 100%, 80–100%, 60–80%, and less than 60%, scored using the Blosum62 matrix. Regions marked "Include" were used to create the phylogenetic tree.

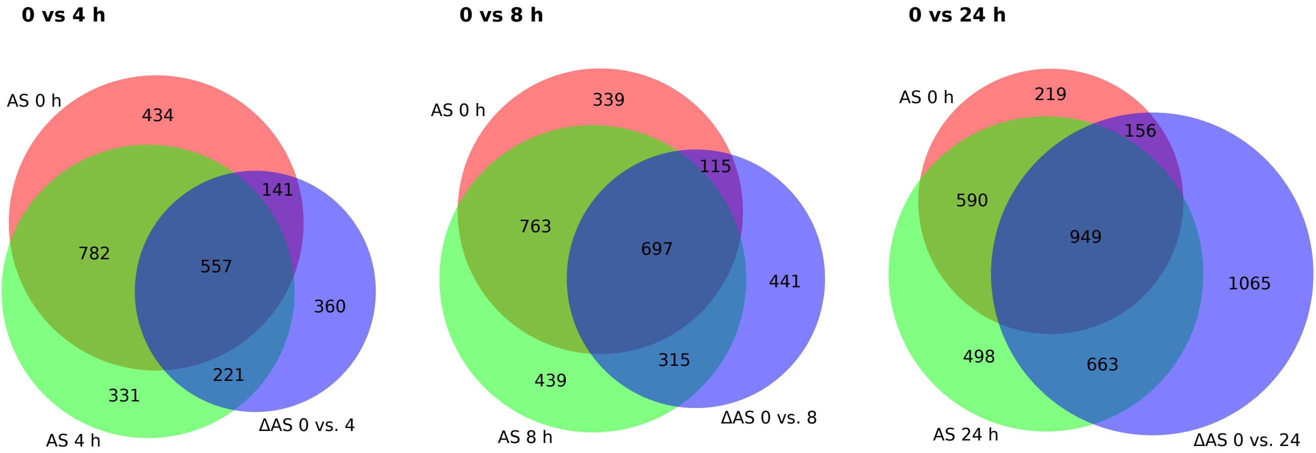

Supplementary Figure S2: Proportional Venn diagrams comparing genes identified as alternatively spliced in a single condition (not including intron retention), versus genes identified as having their alternative splicing changed after overexpression of TgSR3. Red regions represent alternatively-spliced genes in a single, uninduced condition; green regions represent alternatively-spliced genes in a single, induced condition, after 4, 8 or 24 hours overexpression of TgSR3; blue regions represent genes that had their alternative splicing altered between two conditions, after overexpression.
