# Supplementary material for: A serine–arginine-rich (SR) splicing factor modulates alternative splicing of over a thousand genes in Toxoplasma gondii
Source: Nucleic Acids Res. 2015 Apr 13;43(9):4661–75. doi: 10.1093/nar/gkv311 (PMC4482073; doi:10.1093/nar/gkv311)
Supplement: SUPPLEMENTARY DATA [file supp_gkv311_nar-03398-a-2014-File009.docx]

**Supplementary Legends**

**Supplementary Figure S1** - Alignment for phylogeny

**Supplementary Figure S2** - Venn diagrams showing relationship between alternatively-spliced perturbation and alternatively-spliced genes (excluding intron retention)

**Supplementary File S1** - Human gene IDs from phylogeny

**Supplementary File S2** - Primers used

**Supplementary File S3** - RNA-seq analysis pipeline

**Supplementary File S4** - List of differentially-expressed whole genes after 4 hours induction

**Supplementary File S5** - List of differentially-expressed whole genes after 8 hours induction

**Supplementary File S6** - List of differentially-expressed whole genes after 24 hours induction

**Supplementary File S7** - List of genes where alternative splicing has changed after 4 hours induction

**Supplementary File S8** - List of genes where alternative splicing has changed after 8 hours induction

**Supplementary File S9** - List of genes where alternative splicing has changed after 24 hours induction

**Supplementary File S10** - List of alternatively-spliced genes (excluding intron retention) when uninduced

**Supplementary File S11** - List of alternatively-spliced genes (excluding intron retention) at 4 hours

**Supplementary File S12** - List of alternatively-spliced genes (excluding intron retention) at 8 hours

**Supplementary File S13** - List of alternatively-spliced genes (excluding intron retention) at 24 hours
